# Supplementary figures and images for: Transcriptome Analysis of Apples in High-Temperature Treatments Reveals a Role of MdLBD37 in the Inhibition of Anthocyanin Accumulation
Source: Int J Mol Sci. 2022 Mar 29;23(7):3766. doi: 10.3390/ijms23073766 (PMC8998508; doi:10.3390/ijms23073766)

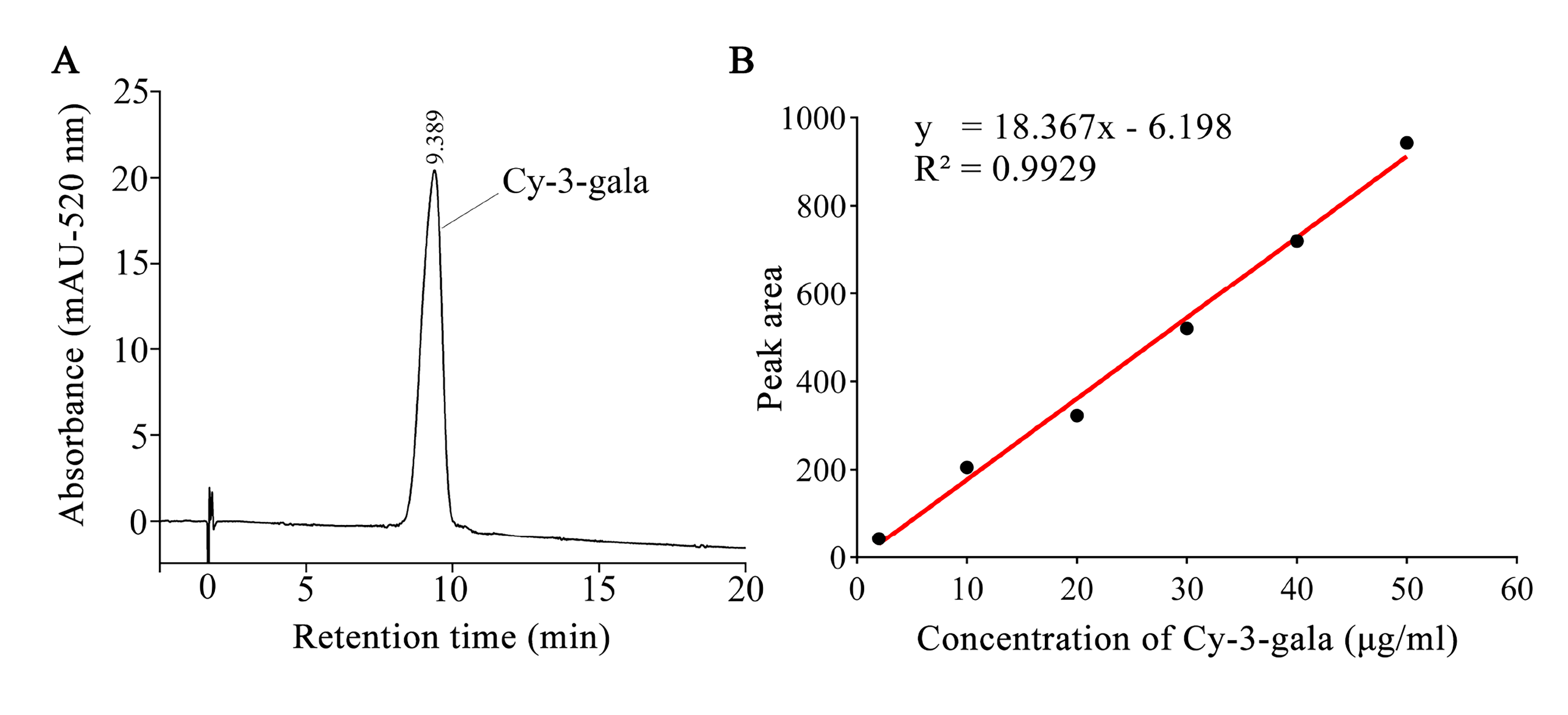

Supplement: Supplementary file 1 [file ijms-23-03766-s001.zip › ijms-1637833 - Supplementary/Figure S1.tif]
